# Hypertension in the Setting of Hypertrophic Obstructive Cardiomyopathy and Cocaine Use

**DOI:** 10.7759/cureus.30090

**Published:** 2022-10-09

**Authors:** Daniel Miller, Asma Hosna, Sanna Salam, Roger Stern, Sanjiv Bakshi

**Affiliations:** 1 Internal Medicine, Icahn School of Medicine at Mount Sinai, Queens Hospital Center, New York, USA; 2 Cardiology, Icahn School of Medicine at Mount Sinai, Queens Hospital Center, New York, USA

**Keywords:** hypertensive urgency, pharmacology, cocaine use, hypertension, hypertrophic obstructive cardiomyopathy (hocm)

## Abstract

Hypertrophic obstructive cardiomyopathy (HOCM) is most commonly an inherited genetic condition where hypertension can be challenging to treat as many antihypertensive medications cannot be used in this patient population. Any agent that decreases preload or afterload should be avoided in this condition, leaving beta-adrenergic receptor antagonists as the preferred agent of choice in these patients. However, a patient with HOCM and cocaine use can pose a significant challenge due to the risks associated with initiating beta-adrenergic receptor antagonists in cocaine users because of the unopposed alpha receptor effect of the treatment, which would in turn cause worsening hypertension. The fact that cocaine itself causes hypertension further complicates the issue. The only remaining class of medications that can be used are non-dihydropyridine calcium channel blockers, which may not be effective on their own against the vasoconstrictive properties of cocaine. Hence, it is paramount to educate all patients with HOCM to avoid cocaine use even more so than other patients.

## Introduction

It can be challenging to treat patients diagnosed with hypertrophic obstructive cardiomyopathy (HOCM) and cocaine use coupled with hypertensive urgency. Generally, there is a robust assortment of medications to choose from and the management can be tailored to the specific risk profiles of each individual patient. A patient who is tachycardic and hypertensive may benefit from a medication that both lowers heart rate and blood pressure, such as a beta-blocker, while one would avoid the use of a direct vasodilatory agent such as hydralazine due to its reflex tachycardia effects in such a patient. We present a case of a patient exhibiting two conditions that left severely limited options to choose from to treat the hypertensive urgency.

## Case presentation

**Figure 1:**
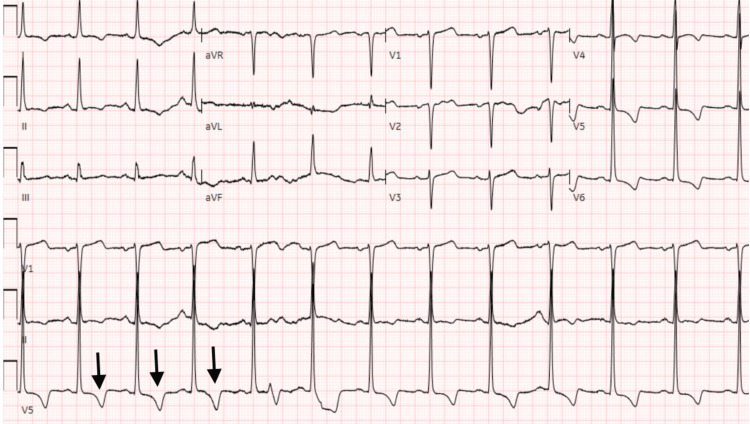
Electrocardiogram Arrows point to T-wave inversions

**Figure 2:**
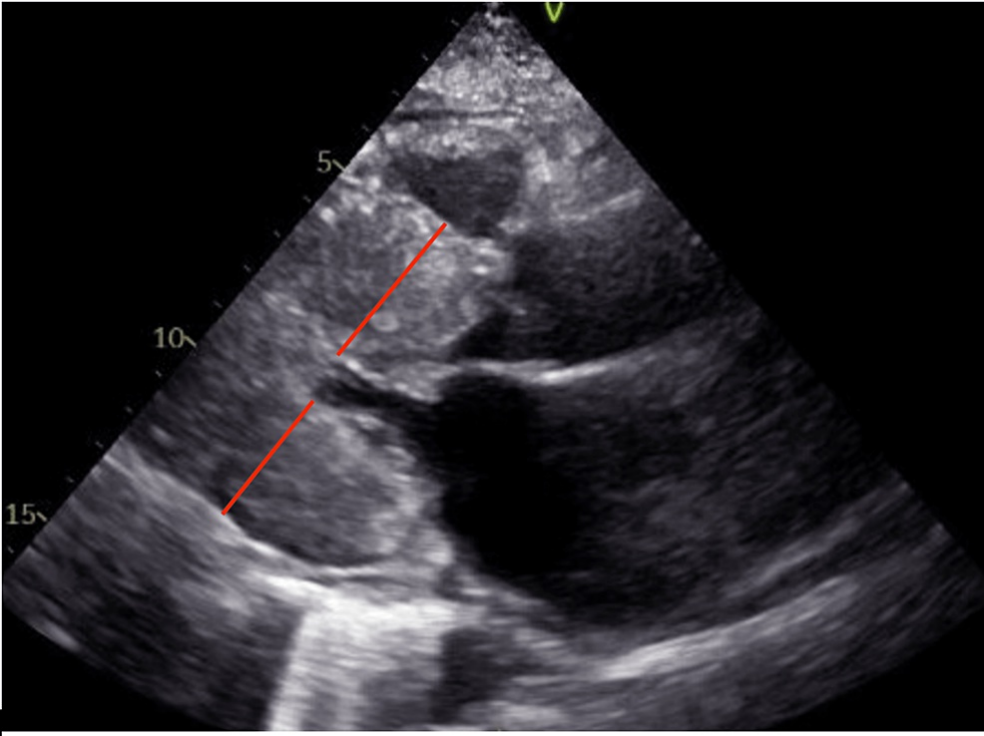
Echocardiogram Red lines show the width of the left ventricular wall

A 60-year-old female patient with a past medical history of HOCM and polysubstance abuse presented to the emergency room with persistent non-radiating chest pain that had started a few hours previously after she consumed some alcoholic beverages. The patient had a heart rate of 99 beats per minute, and a blood pressure of 180/99 mmHg. Her urine test returned positive for cocaine use and the patient was immediately started on diltiazem. Electrocardiogram (EKG) (Figure 1) showed normal sinus rhythm with T-wave inversions in multiple leads. EKG findings were unchanged from the previous one performed eight months prior. Serial troponins were negative. An echocardiogram showed hypertrophic cardiomyopathy with left ventricular outflow obstruction with a systolic anterior motion of the anterior leaflet with a resting gradient of 80 mmHg (normal value: <30 mmHg) (Figure 2).

The patient's blood pressure continued to rise, eventually reaching 197/104 mmHg, despite administering a maximum dose of diltiazem. The benefits and risks of initiating hydralazine were discussed with the patient. Ultimately, the patient agreed to undergo a trial of hydralazine, and the blood pressure came down to 164/80 mmHg without significant side effects from the HOCM. She was discharged with uncontrolled hypertension to follow up as an outpatient for further management and counseling on substance abuse.

## Discussion

HOCM is a cardiac disease that is often associated with a genetic origin [1,2]. The septal wall of the myocardium becomes hypertrophied, thereby causing a systolic anterior motion of the mitral valve leaflet causing the left ventricular outflow tract to be obstructed during high-velocity blood flow from the left ventricle through the left ventricular outflow tract [3]. Unfortunately, there have not been any large randomized trials to treat HOCM, but the consensus entails treating only symptomatic patients [4]. Symptomatic patients can have dyspnea, palpitations, and fatigue, similar to any heart failure patient, due to the congestion caused by blood entering the left side of the heart. However, in stark contrast with heart failure, vasodilatory agents must be avoided in these patients. Some of these agents include dihydropyridine calcium channel blockers and angiotensin-converting enzyme inhibitors. This is because the less resistance the heart faces in pumping the blood forward, the more velocity the blood has, thereby causing the anterior mitral leaflet to obstruct the outflow tract even more. Another factor to consider is the end-diastolic volume. The greater the left ventricular end-diastolic volume, the lower the outflow obstruction. It is assumed that more volume in the heart leads to more septum being pushed away from the outflow tract. The goal in treating symptomatic HOCM is to decrease the chronotropic response of the heart to allow for greater end-diastolic volume and to ensure that there is enough preload for the same reason. Additionally, afterload should not be decreased as it will increase the velocity of the forward flow, which would increase the left ventricular outflow obstruction. Hence, diuretics are usually avoided in these patients.

Hypertension in the setting of cocaine use must be managed carefully as some commonly used antihypertensives can actually exacerbate the issue. Cocaine is thought to be an alpha as well as a beta-agonist. Thus, if one is given a beta-blocker, it will in turn cause an unopposed alpha response constricting arterioles and increasing hypertension [5].

**Figure 3:**
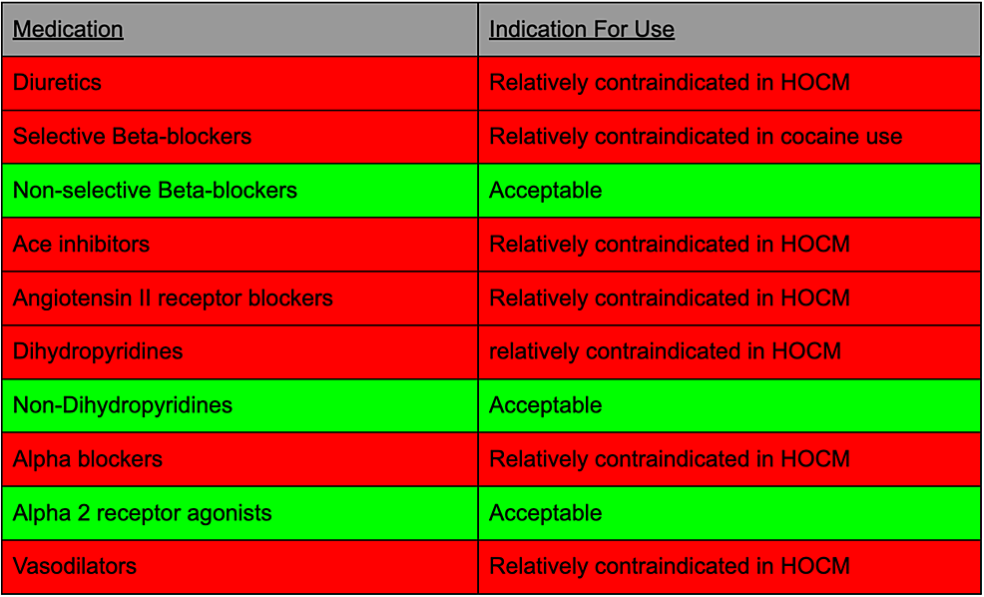
Medication options and their Indications Red indicates relative contraindication. Green indicates no contraindication HOCM: hypertrophic obstructive cardiomyopathy

Currently, in treating a patient presenting with HOCM and cocaine use with hypertension, there is a very limited option of medications to choose from (Figure 3) [6,7].

## Conclusions

Patients with HOCM who present with hypertension must be carefully evaluated before antihypertensive medications are administered. Medications that decrease afterload or preload should be avoided, limiting the use of many antihypertensive agents in this patient population. The medication of choice in these patients is beta-adrenergic receptor antagonists. However, if the patient has had recent cocaine use, beta-adrenergic receptor antagonists should be avoided as well to prevent the risk of uninhibited alpha-adrenergic effects. However, non-selective beta-blockers can be used along with non-dihydropyridine calcium channel blockers to control hypertension. Nevertheless, any patient who is diagnosed with HOCM should be made aware of the extra risks and detrimental effects of cocaine use in this population group.
